# Chromosomal assembly of the nuclear genome of the endosymbiont-bearing trypanosomatid *Angomonas deanei*

**DOI:** 10.1093/g3journal/jkaa018

**Published:** 2020-11-27

**Authors:** John W Davey, Carolina M C Catta-Preta, Sally James, Sarah Forrester, Maria Cristina M Motta, Peter D Ashton, Jeremy C Mottram

**Affiliations:** 1 Department of Biology, University of York, York YO10 5DD, UK; 2 York Biomedical Research Institute, University of York, York YO10 5DD, UK; 3 Medicinal Chemistry Center (CQMED)/Structural Genomics Consortium, Universidade Estadual de Campinas, Campinas, São Paulo 13083-886, Brazil; 4 Laboratório de Ultraestrutura Celular Hertha Meyer, Instituto de Biofísica Carlos Chagas Filho, Departamento de Biologia Celular e Parasitologia, Centro de Ciências da Saúde, Universidade Federal do Rio de Janeiro, Rio de Janeiro, RJ, Brazil; 5 Instituto Nacional de Ciência e Tecnologia em Biologia Estrutural e Bioimagens, Rio de Janeiro, RJ, Brazil

**Keywords:** *Angomonas deanei* Carvalho (ATCC^®^ PRA-265™), Oxford Nanopore, genome assembly

## Abstract

*Angomonas deanei* is an endosymbiont-bearing trypanosomatid with several highly fragmented genome assemblies and unknown chromosome number. We present an assembly of the *A. deanei* nuclear genome based on Oxford Nanopore sequence that resolves into 29 complete or close-to-complete chromosomes. The assembly has several previously unknown special features; it has a supernumerary chromosome, a chromosome with a 340-kb inversion, and there is a translocation between two chromosomes. We also present an updated annotation of the chromosomal genome with 10,365 protein-coding genes, 59 transfer RNAs, 26 ribosomal RNAs, and 62 noncoding RNAs.

## Introduction

*Angomonas deanei* is a trypanosomatid that mutually coevolves with an endosymbiont, a β-Proteobacterium of the Alcaligenaceae family that contains a reduced genome when compared to its ancestral prokaryote. The symbiont divides during the host cell cycle such that each new protozoan contains a single bacterium. Trypanosomatid endosymbiosis involves an intense metabolic exchange: the bacterium supplies the protozoan with amino acids, heme, and vitamins, while benefiting from the host’s energy and phospholipid production (de Azevedo-Martins *et al.* 2007; Motta *et al.* 2010; Alves *et al.* 2011, 2013; Klein *et al.* 2013; Loyola-Machado *et al.* 2017). Thus, endosymbiosis in trypanosomatids has been used to study cell evolution and the origin of organelles.

Symbiont-harboring trypanosomatids are distributed in four genera: *Angomonas*, *Strigomonas* (Teixeira *et al.* 2011), and *Kentomonas* (Votýpka *et al.* 2014), constituting the Strigomonadinae subfamily, and the phylogenetically distant genus *Novymonas* (Kostygov *et al.* 2016). They have ultrastructural and biochemical features that distinguish them from other monoxenics and human pathogenic trypanosomatids, such as *Trypanosoma cruzi* and *Leishmania* sp., the latter a phylogenetically close genus to symbiont-harboring trypanosomatids. While draft genome assemblies are available for *Angomonas* and *Strigomonas*, there are no complete chromosomal assemblies for any of the four genera of symbiont-harboring trypanosomatids.

The first genome sequencing of *A. deanei* identified the predicted proteins of the protozoan and its symbiont (Motta *et al.* 2013), and two further sequencing efforts have produced fragmented whole-genome assemblies (Alves *et al.* 2013; Morales *et al.* 2016). These assemblies have been used to study the loss, transference, and interference of genes during symbiosis (Alves *et al.* 2013), as well as to investigate heterologous or endogenous gene and protein expression (Catta-Preta *et al.* 2016; Morales *et al.* 2016; Penha *et al.* 2016). However, the structure and full noncoding regions of the genome have not been resolved yet. Here, we present a new assembly of the *A. deanei* genome, sequenced using Oxford Nanopore single-molecule technology, which is resolved into 29 chromosomes and reveals several previously unknown special features of the genome. We expect that the new assembly will assist future studies of symbiont-harboring trypanosomatids and other trypanosomatids and monoxenics.

## Materials and methods

Supplementary methods and Supplementary file descriptions can be found in Supplementary File S1.

### Organism/strain origin and derivation

*Crithidia deanei* Carvalho (ATCC^®^ PRA-265™) (Carvalho 1973), now *A. deanei* (Teixeira *et al.* 2011) was cultivated axenically in Warren’s medium (Warren 1960), supplemented with 10% fetal calf serum for 24 h at 27°C. Cells were concentrated to 10^8^ by centrifugation (×1200 *g* for 10 min) before DNA extraction.

### Sample preparation

DNA was extracted from snap-frozen pellets containing approximately 10^8^ cells using a beta version of the Nanobind CBB Big DNA Kit (Circulomics Inc.), according to the manufacturer’s guidelines, using the HMW protocol for gram-negative bacteria. Briefly, cell pellets were resuspended in 20-μl PBS before addition of equal volumes of proteinase K and kit cell lysis buffer CLE, and incubation at 55°C for 20 min. Samples were then treated with RNase A for 5 min at room temperature, before the addition of kit buffer BL3 and a further 15-min incubation at 55°C. DNA was precipitated with isopropanol, in the presence of the Nanobind disk, washed as per the protocol, and eluted from the disk into Tris elution buffer. DNA was left overnight at 4°C to fully resuspend before further processing.

### Sequencing

For high accuracy short-read sequencing, a paired-end library was prepared using the NEBNext Ultra II FS DNA library prep kit for Illumina (New England Biolabs), according to the manufacturer’s instructions, using 100 ng starting DNA, and using four cycles of PCR amplification using NEBNext multiplex oligos for Illumina (unique dual index primers; NEB). The library was then subject to 2 × 150 bp sequencing on an Illumina HiSeq 3000 sequencer, at the University of Leeds Next Generation Sequencing Facility.

Long-fragment DNA sequencing was performed using an Oxford Nanopore Technologies (ONT) MinION sequencer. Approximately 500-ng genomic DNA was subject to shearing using the Covaris g-TUBE™ to a mean fragment size of 20 kb, and mixed with an additional 1 µg of unsheared genomic DNA. The sequencing libraries were generated using the SQK-LSK109 ligation sequencing kit (ONT). Library preparation started with DNA repair/A-tailing using the NEBNext^®^ Ultra™ II End Repair/dA-Tailing Module, with additional NEBNext FFPE repair enzyme (NEB), using sequential incubations for 30 min at 20°C and then 65°C. Following clean up with 0.9× volume AMPure XP beads (Beckman Coulter), adapters were ligated to prepared DNA ends using NEBNext quick T4 DNA ligase, and the ligation buffer provided in the SQK-LSK109 kit. An additional clean up with AMPure XP beads, including two washes using the ONT Long Fragment Buffer, was performed prior to elution into the buffer provided. The total eluted library was then loaded onto an ONT FLO-MIN109 R9.4.1 flow cell, following the manufacturer's guidelines, and run for 48 h using MinKNOW software.

### Sequence processing and genome assembly

Oxford Nanopore MinION sequences were base called with Guppy 3.1.5. Adapters were trimmed using Porechop 0.2.3 (https://github.com/rrwick/Porechop). The raw MinION reads were assembled with Canu 1.8 (Koren *et al.* 2017) (https://canu.readthedocs.io) with option “genomeSize = 23m.” The raw assembly (Supplementary File S2) was manually assessed and edited (see Supplementary File S1 Section 1, File S3, and File S4 for full details). Contig and read alignments for assessments were produced with minimap2 2.17-r941 (Li 2018) (https://github.com/lh3/minimap2) and were inspected using IGV 2.5.3 (Thorvaldsdóttir *et al.* 2013) (http://software.broadinstitute.org/software/igv/). The genome was edited with seqkit 0.10.0 (Shen *et al.* 2016) (https://bioinf.shenwei.me/seqkit/).

The filtered assembly was polished with medaka 0.7.1 (https://github.com/nanoporetech/medaka), using a filtered set of reads longer than 20 kb generated with filtlong 0.2.0 (https://github.com/rrwick/Filtlong) using options min_length 20000 and keep_percent 90. The medaka-polished assembly was polished with Illumina data three times using Pilon 1.22 (Walker *et al.* 2014) (https://github.com/broadinstitute/pilon) (Supplementary File S5 contains the polished assembly). Before polishing, Illumina sequences were adapter trimmed with cutadapt 1.16 (Martin 2011) (https://cutadapt.readthedocs.io) for the Illumina Universal Adapter sequence AGATCGGAAGAG. bwa 0.7.17 (Li 2013) (https://github.com/lh3/bwa) and samtools 1.9 (Li *et al.* 2009) (http://www.htslib.org) were used to align Illumina reads to the assembly for polishing.

### Validation with PCR

PCRs to validate assembly features (Figure 2; see Supplementary File S1 Section 2.2 for further details) were prepared with 10 ng of *A. deanei* DNA in each reaction (or water for negative controls), mixed with a low ROX SYBR Green master mix and run on a QuantStudio 3, using a 2 step fast PCR with a 2 s denaturing step at 95°C and 30 s anneal and extend step at 60°C, for 32 cycles. Fifteen microliters of each product was run on a 2% agarose gel with an Invitrogen 50-bp DNA ladder.

### Annotation

The previous genome annotation (Motta *et al.* 2013), NCBI accession GCA_000442575.2, was transferred with flo (Pracana *et al.* 2017). Duplicate annotations and erroneous proteins were fixed with a custom Python script (Supplementary File S6; output in Supplementary File S7), and the genome was also annotated using Companion version 1.0.2 (Steinbiss *et al.* 2016) (Supplementary Files S8–S12). Full details of the annotation process are in Supplementary File S1 Section 3. Median TriTrypDB statistics were calculated by downloading a table of genome information from https://tritrypdb.org (downloaded on December 11, 2019 via Data Summary → Genomes and Data Types; on January 6, 2021 the same data was available via Data → Organisms: Genome Info & Stats) and restricting to reference genomes only.

### Genome analysis

**Figure 1:**
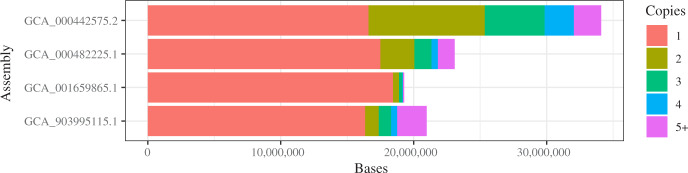
Redundancy of genome assemblies. Bars show number of bases in assemblies colored by copy number. Unique material has only one copy in the assembly (red). Highly repetitive material has many copies. Large amounts of material with two or three copies suggest haplotypic variation has been retained, although some nonunique material is expected due to common repeats.

Redundancy of genome assemblies (Figure 1) was assessed by aligning genomes to themselves with minimap2 2.17-r941 (Li 2018) using options -x ava-ont and -a to output SAM format; alignments were then sorted and indexed with samtools 1.9 (Li *et al.* 2009). Copy numbers were calculated with mosdepth 0.2.5 (Pedersen and Quinlan 2018) (https://github.com/brentp/mosdepth) using option -F 0 to include all alignments. A script (Supplementary File S13) was run to calculate the number of bases assigned to each copy number from the mosdepth output. chr02 was identified as supernumerary (Figure 3) by aligning *A. deanei* Illumina reads used for polishing to the polished assembly with bwa 0.7.17 (Li 2013) and calling variants with freebayes v1.1.0-3-g961e5f3 (Garrison and Marth 2012) (https://github.com/ekg/freebayes) with option -F 0 to accept variants with any alternate fraction. The freebayes VCF was filtered to heterozygote SNPs only using perl and awk, and filtered to only unique regions of the genome using bcftools 1.9 (Li 2011) (https://www.htslib.org) and the mosdepth BED file from the genome redundancy analysis (see above).

Assemblies were assessed with BUSCO v4.0.6 (Seppey *et al.* 2019) (https://busco.ezlab.org) using lineage eukaryota_odb10 and options -m genome and long.

### Data availability

Raw reads are available in the European Nucleotide Archive (project, PRJEB36170; study, ERP119328; sample, ERS4235756; Oxford Nanopore reads, ERR3813852; Illumina reads, ERR3813853). The assembly and annotation are available at accession GCA_903995115; chromosome sequences, LR877145-LR877173. All URLs were accessible on 6 January 2021. Supplemental material is available at figshare DOI: https://doi.org/10.25387/g3.13252664.

## Results and discussion

The genome size and chromosome number for *A. deanei* are unknown. Three previous genome assemblies are available (Table 1) (Alves *et al.* 2013; Motta *et al.* 2013; Morales *et al.* 2016). The first (Motta *et al.* 2013)is a reference-guided assembly aimed at identifying protein-coding gene sequences, using a set of 73.8 thousand protein sequences from TriTrypDB 3.3 as a reference, but also including contigs assembled from reads that did not align to the reference. All three assemblies are fragmented and two contain many gaps. They are also of varying sizes (34.1, 23.1, and 19.3 Mb). However, the first assembly contains only 16.6 Mb of unique material, with a further 13.2 Mb of sequence occurring two or three times in the genome (Figure 1). Nonunique material may be accurate expansions of highly repetitive sequence, but could also be extra haplotypic material that should be removed. Of 129 complete eukaryotic BUSCOs found in this assembly, 88 (68% of complete BUSCOs) are duplicated (Table 1). This suggests the first assembly contains many haplotypic sequences, not found to such an extent in the other assemblies, and so the true genome size is likely to be closer to 20 Mb than 35 Mb.

**Table 1:** Summary of *A. deanei* genome assemblies

NCBI ID	GCA_000442575.2	GCA_000482225.1	GCA_001659865.1	GCA_903995115.1
Name	Angomonas_deanei_Genome	Adea_1.0	Angomonas_deanei_v1.0	Adeanei_nanopore_chromosomes
Reference	Motta *et al.* (2013)	Alves *et al.* (2013)	Morales *et al.* (2016)	This paper
Scaffolds	17 339	5 616	408	29
Length (bp)	34 103 807	23 079 371	19 282 250	20 976 081
Scaffold N50	2 497	11 595	300 798	774 942
Gaps (bp)	30 204	197	1 728 731	0
Complete BUSCOs	129 (50.6%)	125 (49.1%)	127 (49.8%)	128 (50.2%)
Complete, single-copy BUSCOs	41 (16.1%)	120 (47.1%)	124 (48.6%)	125 (49.0%)
Complete, duplicated BUSCOs	88 (34.5%)	5 (2.0%)	3 (1.2%)	3 (1.2%)
Fragmented BUSCOs	22 (8.6%)	21 (8.2%)	20 (7.8%)	21 (8.2%)
Missing BUSCOs	104 (40.8%)	109 (42.7%)	108 (42.4%)	106 (41.6%)

BUSCO statistics are from a set of 255 eukaryotic benchmarking universal single-copy orthologs (BUSCOs) (Seppey *et al.* 2019). Percentages are calculated from all 255 BUSCOs.

We sequenced 2,051,753 Oxford Nanopore MinION reads containing 13,302,088,880 bp of sequence after adapter trimming (665 times coverage of a 20 Mb genome) with a read N50 of 14,610 bp, and 9,775,722 Illumina HiSeq 3000 read pairs totaling 2,952,268,044 bp (read length 150 bp, 148 times coverage of a 20 Mb genome). We assembled the MinION sequence with Canu (Koren *et al.* 2017) to produce an initial raw genome assembly containing 212 contigs, 27,914,394-bp long (Supplementary File S2), with a contig N50 of 646,966 bp and no gaps, already an improvement on any existing assembly.

To improve the raw Canu assembly (Supplementary File S2), we ran the assembly through Tapestry (Davey *et al.* 2020) (https://github.com/johnomics/tapestry) to calculate quality information for each contig (Supplementary Table S1, File S3), and then filtered and edited the genome based on this information (Supplementary Table S1, File S1 Section 1, File S4, Figures S1–S13). The assembly contained a symbiont genome in 1 contig (Supplementary File S1 Section 1.1), 127 contigs from the kinetoplast minicircle (which were removed from the assembly; Supplementary File S1 Section 1.2) (Lukeš *et al.* 2002), and 3 contigs from the kinetoplast maxicircle (which were reduced to one unique copy of the maxicircle) (Supplementary File S1 Section 1.3, Figure S1). As full-length accessory genomes are already publicly available [symbiont: NCBI GenBank GCA_000319225.1 (Motta *et al.* 2013) and GCF_000340825.1 (Alves *et al.* 2013), maxicircle: NCBI GenBank KJ778684.1], these have been removed from our public assembly (NCBI GenBank GCA_903995115), but they are available in our polished assembly included with this paper (Supplementary File S5).

**Figure 2:**
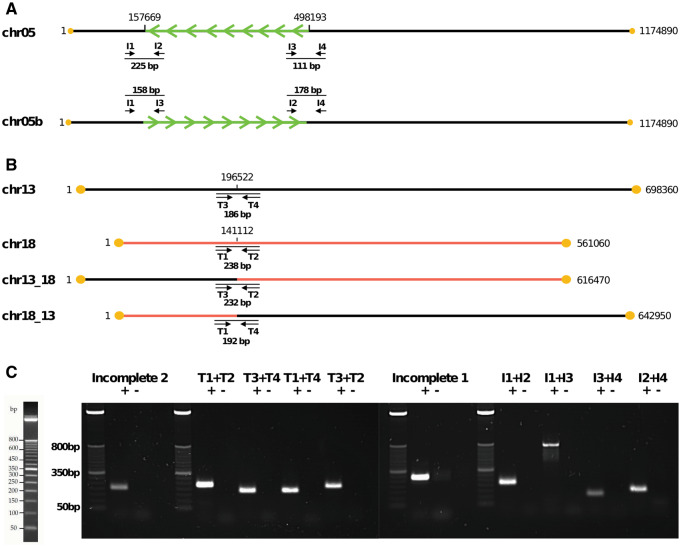
PCR validation of special features. (A) Chromosome 5 inversion. Inversion shown with arrows in green. Primers I1, I2, I3, and I4 were designed to span the breakpoints of the two inversion haplotypes chr05 and chr05b. Primer products shown as thin black lines (not to scale); expected primer product size is shown for each primer pair. Breakpoint positions in polished genome (Supplementary File S5) given above chr05. Yellow dots are telomeres. (B) Chromosome 13/18 translocation. Primers T1, T2, T3, and T4 were designed to span the breakpoints of the four translocation haplotypes chr13, chr18, chr13_18, and chr18_13. Key as in Figure 2A. (C) PCR products shown via gel electrophoresis against a 50-bp Invitrogen DNA ladder (left) for the inversion (I1–I4), the translocation (T1–T4) and two incomplete chromosomes (Supplementary File S1 Section 2.2, File S5, Table S3 and S4). “+” and “−” lanes show product and negative control (water), respectively.

This left 81 contigs from the nuclear genome. Of these, 49 contigs were extra repeat copies, subtelomeric, or haplotypic and were removed from the assembly, leaving 32 contigs (see Supplementary Table S1 for details). Manual inspections resolved these contigs to 29 complete or close-to-complete chromosomal sequences, with incomplete contigs explainable due to a translocation (Supplementary Figures S2 and S3), an inversion (Supplementary Figure S4), and several misassemblies (Supplementary Figures S5–S10) (all discussed in detail in Supplementary File S1 Sections 1.4–1.8; genome edits and translocation and inversion haplotypes summarized in Supplementary Table S2). Fifty-six of 58 contig ends have multiple copies of the trypanosome telomere sequence CCCTAA/TTAGGG (Dreesen *et al.* 2007); although the remaining two contig ends do not contain telomeres, the majority of reads that align to these ends do contain telomeres, so these ends are likely to be almost complete (Supplementary File S1 Section 1.9, Figures S11–S13). The translocation and inversion were validated with read alignments (Supplementary File S1 Section 2.1, Table S2, Figures S14–S21) and with PCR (Figure 2, *Materials and methods*, Supplementary File S1 Section 2.2, Tables S3 and S4).

We transferred the first *A. deanei* gene annotation (NCBI genome GCA_000442575.2) to our new nuclear genome assembly, and also predicted new genes and RNAs where possible (see *Materials and methods*, Supplementary File S1 Section 3, Supplementary Files S6–12). The new annotation has 10,365 protein-coding genes (7199 transferred, 3166 newly predicted), 59 tRNAs covering all 20 standard amino acids and 1 tRNA for selenocysteine, 26 ribosomal RNAs, and 62 noncoding RNAs (45 ncRNA, 14 snoRNA, 3 snRNA). This compares well to other reference genomes in the Kinetoplastid Genomics database TriTrypDB, which have median 8652 protein-coding genes (median absolute deviation 387) and 110 nonprotein coding genes (median absolute deviation 27).

**Figure 3:**
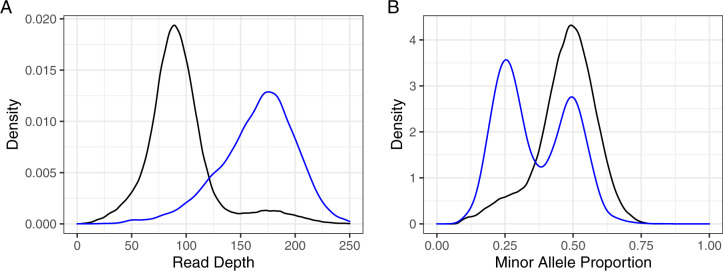
Chromosome 2 is a supernumerary chromosome. (A) Read depths at SNPs in unique regions across the whole nuclear genome (black) or on chr02 only (blue). Chr02 median depth (170) is roughly double the whole-genome median depth (88), indicating chr02 may have double the copy number of the rest of the genome. (B) Proportion of reads with minor allele for all SNPs in unique regions across the genome (black) or on chr02 only (blue). 0.5 (1:1 ratio) indicates two copies; a mixture of 0.25 (1:3 ratio) and 0.5 indicates four copies.

**Figure 4:**
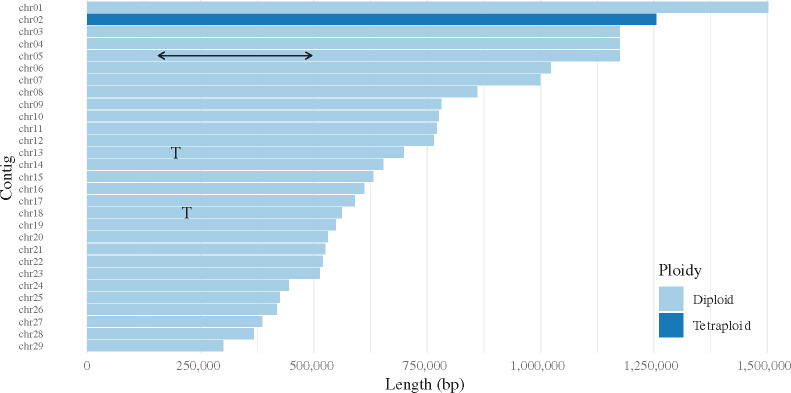
Chromosome lengths in new *A. deanei* nuclear genome assembly. chr02 is supernumerary (dark blue), chr05 has a 340-kb inversion (line with arrows), and chromosomes 13 and 18 translocate at the points marked “T.”

We therefore propose that *A. deanei* has 29 chromosomes, and have named the remaining 29 contigs chr01 to chr29 in order of size, largest first (Supplementary File S9). These 29 chromosomes make a nuclear genome of 20,976,081 bp, chromosome N50 774,942 bp, with no gaps (Supplementary Table S5). The assembly has a supernumerary chromosome, in common with other trypanosomatids (Downing *et al.* 2011; Rogers *et al.* 2011; Reis-Cunha *et al.* 2018), with chromosome 2 (chr02) having considerably higher read depth than other chromosomes (Figure 3A); the contig has a mixture of 1:1 and 3:1 ratios for SNP calls (Figure 3B), which suggests there are four copies of this chromosome, not two, as for the remaining diploid chromosomes. There is an inversion on chromosome 5 (chr05) between 157.6 and 498.1 kb, 340.5-kb long (1.61% of the nuclear genome), containing 173 genes (1.67% of the protein-coding genes in the nuclear genome). Chromosomes 13 and 18 (chr13, chr18) reciprocally translocate at chromosome 13 196.6 kb and chromosome 18 141.1 kb. Figure 4 shows the genome with these features; the lengths of the contigs are summarized in Supplementary Table S5.

All four public *A. deanei* genome assemblies have very similar BUSCO scores (Table 1), indicating that all four assemblies have similar gene coverage, despite the excess of duplicated genes in the GCA_000442572.2 assembly. The low yet consistent percentages of eukaryotic BUSCO genes across all *A. deanei* assemblies suggest this eukaryotic gene set is not representative of the *A. deanei* genome, rather than suggesting a large number of *A. deanei* genes are missing from all of these assemblies; nevertheless, the BUSCO gene set is useful for comparing the four assemblies. Our new assembly matches the gene coverage of the other assemblies, with slightly higher complete single-copy gene sequences, while greatly improving genome contiguity.

We expect our new high-quality, close-to-complete genome assembly, including full chromosome sequences and many noncoding RNAs and nongenic regions, will be useful for future research. It is the first chromosomal assembly for any endosymbiont-bearing trypanosomatid. MicroRNAs have been reported as important regulators of symbiosis in plants (Hussain *et al.* 2018; Hossain *et al.* 2019), an interesting mechanism that can now be investigated in *A. deanei*, the model of symbiosis in trypanosomatids. Recently, a Brazilian patient presenting symptoms of leishmaniasis was nonresponsive to available treatments and was found to be infected with a new trypanosomatid phylogenetically related to *Crithidia fasciculata*, a monoxenic trypanosomatid for which only an unpublished draft genome is available (Maruyama *et al.* 2019). There are few monoxenic genomes that can be used as a reference in such cases, as well as in coinfections of pathogens and the so-called nonpathogens (Pacheco *et al.* 1998; Srivastava *et al.* 2010; Ghosh *et al.* 2012; Kraeva *et al.* 2015). This new *A. deanei* assembly can now be used to assist in the identification of new, possibly pathogenic, species. Moreover, a toolkit for reverse genetic studies is being developed for *A. deanei*, which will illuminate more of the biology of the protozoan and its symbiotic relationship with a prokaryote, and the evolutionary leap from symbiont to organelle. Finally, the assembly provides another example of small genomes being almost completely resolved with single runs of long-read sequencing, with close examination of the sequences revealing special features of the genome never known before.
